# Molecular Evidence that Only Two Opsin Subfamilies, the Blue Light- (SWS2) and Green Light-Sensitive (RH2), Drive Color Vision in Atlantic Cod (*Gadus morhua*)

**DOI:** 10.1371/journal.pone.0115436

**Published:** 2014-12-31

**Authors:** Ragnhild Valen, Rolf Brudvik Edvardsen, Anne Mette Søviknes, Øyvind Drivenes, Jon Vidar Helvik

**Affiliations:** 1 Department of Biology, University of Bergen, High Technology Centre, N-5020, Bergen, Norway; 2 Institute of Marine Research, P.O. Box 1870, Nordnes, 5817, Bergen, Norway; University Zürich, Switzerland

## Abstract

Teleosts show a great variety in visual opsin complement, due to both gene duplication and gene loss. The repertoire ranges from one subfamily of visual opsins (scotopic vision) including rod opsin only retinas seen in many deep-sea species to multiple subfamilies of visual opsins in some pelagic species. We have investigated the opsin repertoire of Atlantic cod (*Gadus morhua*) using information in the recently sequenced cod genome and found that despite cod not being a deep sea species it lacks visual subfamilies sensitive towards the most extreme parts of the light spectra representing UV and red light. Furthermore, we find that Atlantic cod has duplicated paralogs of both blue-sensitive SWS2 and green-sensitive RH2 subfamilies, with members belonging to each subfamily linked in tandem within the genome (two SWS2-, and three RH2A genes, respectively). The presence of multiple cone opsin genes indicates that there have been duplication events in the cod ancestor SWS2 and RH2 opsins producing paralogs that have been retained in Atlantic. Our results are supported by expressional analysis of cone opsins, which further revealed an ontogenetic change in the array of cone opsins expressed. These findings suggest life stage specific programs for opsin regulation which could be linked to habitat changes and available light as the larvae is transformed into an early juvenile. Altogether we provide the first molecular evidence for color vision driven by only two families of cone opsins due to gene loss in a teleost.

## Introduction

### Principles, limitation and evolution of vision in the ocean

The optical properties of the water column which fish inhabits are dramatically influenced by light intensity and the relative absorption of light by the water itself. While the oceanic water appears blue due to poor levels of nutrient and thus transmits more light, the coastal waters and freshwaters appear greener in color due to higher absorption of short-wavelength light by phytoplankton [1]. However, most of the light of shorter and longer wavelengths are limited to the upper levels of the water column, while blue light is able to penetrate deepest, which in the latter case necessitates scotopic adapted vision [2]. The consequence is a wide range of visual adaptations with varying degree of light absorbing capabilities.

Although there is variation in eye anatomy and physiology, -and in the opsin gene repertoire among marine fishes, the principles of optimal visual perception is to catch and absorb available photons that allows formation of the best possible image in terms of contrast, movement and depth [2]. Moreover, color vision adds further complexity to vision and improves the perception of the environment. In the early vertebrates, color vision provided a mechanism for detecting a possible predator or prey against its background in shallow waters with unfavorable flickering of illumination [3]. In order to discriminate between colors, the early vertebrate eye had to separate and compare different wavelength bands of the light spectra, which resulted in the divergence of UV short wave visual pigments from the common ancestor of long-wave visual pigments [3], [4]. Although color vision increases chromatic perception, resolution or acuity may be diminished due to the extra space needed for multiple classes of photoreceptor cells within the retina[2]. Consequently, the driving force for eye evolution is the combined capacity of color discrimination while at the same time balancing the tradeoff between resolution and chromatic sensitivity [5].

### Photoreceptor mechanism and evolution

It is the visual pigments in the outer segments of retinal rod and cone photoreceptor cells that absorb photons and transfer the information of an image into neuronal signals conveyed to the brain. The visual pigments consist of an opsin protein moiety belonging to the family of G-protein-coupled receptors, and a chromophore of either 11-cis retinal or 11-cis 3,4-dehydroretinal in vertebrates [6]. The photo transduction cascade is initiated when a photon isomerize the covalently bound 11-cis retinal chromophore to all-trans retinal, resulting in a structural change which activates the opsin [7]. The visual pigments are classified according to the specific type of cone photoreceptor cell in which they are expressed. The traditional view has been one class of opsin in each distinct type of cone. In vertebrates, cone opsins are classified into four phylogenetic groups: Ultraviolet-blue or short-wave-sensitive-1-cone-opsin group (SWS1), blue or short-wave-sensitive-2-cone-opsin group (SWS2), green or rod-opsin-like cone-opsin group (RH2) and red-green or long-to-middle-wave-sensitive cone-opsin group (LWS/MWS) [7].

Studies on visual opsin evolution, including analysis of conserved synteny indicate that while a local duplication produced *LWS and SWS*, the subsequent 2R whole genome duplication event expanded the visual opsins into five subfamilies of visual opsins early in vertebrate evolution, and close to appearance of jaws (gnathostome ancestor) [4], [8]-[10]. Furthermore it is suggested that SWS gave rise to SWS1, SWS2, RH1 (rhodopsin) and RH2, and LWS produced four copies however only one was retained [8]. In teleost fishes there are several paralogs within each opsin subfamily, and phylogenetic studies have suggested that these paralogs are mainly a result of tandem duplications that have accumulated over the past 250 million years, rather than from whole genome duplication events [11].

The sum of more recent duplicates combined with retention of old paralogs have produced the large opsin repertoire reported in teleost fishes [11]. Together with variation in selective pressure, favorable mutations at specific amino acid positions may have gained new spectral properties or changed expression patterns (neofunctionalization or subfunctionalization), that may explain the divergence of teleost retinas ranging from scotopic rod opsin only retinas in many deep-sea species to multiple chromatic sensitivity in some pelagic species [12], [13].

### Biology of Atlantic cod

The Atlantic cod, *Gadus morhua*, is a commercially important demersal benthopelagic teleost with a habitat ranging from the shoreline to the continental shelf in the North Atlantic and adjacent seas [14]. The cod has three ontogenetic life stages including an embryonic, larval and juvenile/adult stage. Each life stage is characterized by distinct changes in morphology and ecology and the latter involves metamorphosis of cod larva into juveniles [15]. Cod are highly fecund seasonal batch spawners and the female cod produces up to 300,000 eggs per batch and up to 19 batches per season [16]. The spawning season for Atlantic cod varies geographically between January to May [17]. The fertilized cod eggs and hatched larvae are found in the subsurface bright light scotopic oceanic layers, while the juvenile cod settles in photopic and scotopic deeper waters within its first year [18], [19]. Although microspectrophotometric (MSP) analysis was performed on adult cod more than two decades ago, no molecular characterization of cod visual pigments and photoreceptor organization has yet to be described [20].

### Development of vision in marine fish larvae and aim of study

Studies on retina of marine fish during development typically show an indirect developmental pattern, with only cone driven vision during the larval stages, while rods appear at a later stage usually around the time of metamorphosis [21]. Examples of such species includes; herring; *Clupea harengus* [22], perch, *Perca fluviatilis* [23], greenback flounder, *Rhombosolea tapirina* [24], blackbream, *Acanthopagrus butcheri* [25], red seabream, *Pagrus major* [26], New Zealand snapper, *Pagrus auratus* [27] and Atlantic halibut, *Hippoglossus hippoglossus* [28], [29]. In contrast to the aforementioned species, the Atlantic tarpon (*Megalops atlanticus*) shows an opposite retinal development where the larval retina consist of rods only, then later develops cones [30].

Fishes show a great degree of ontogenetic plasticity in both the complement of cone opsins expressed and the relative number of specific cones expressed within the retina [31], [32]. Several studies show that visual pigments within the same cone opsin family, or even whole families, may be activated or inhibited during development [30], [33]-[36]. Altogether these studies suggest that the external factors influencing changes in retinal sensitivity during development, most likely involves a combination of change in habitat, prey type, mate choice and available light, all acting on the internal array of available opsins. Regardless of numerous studies on cone driven color vision in freshwater species such as, zebrafish, *Danio rerio* [37], goldfish, *Carassius auratus* [38], and a number of cichlids [39], the dynamics of photoreceptor topographic patterns in retina and functional significance of the various visual pigments during development of marine fish larva have got little attention in the literature. There is therefore a gap in our understanding of how the larval visual system of different species is adapted to the light environment they reside, in order to optimize feed and avoid predators. To date, there are limited studies that characterize the genomic arrangement of visual pigments in combination with life stage specific activity [40], [41]. High throughput genome sequencing projects also on marine fish species enables a total overview of visual pigments present in the genome. In the future this information can be used to analyze the activity of these genes under different life stages and environments.

Genome sequencing has allowed a complete overview of all visual pigments present in the species, which represents the repertoire available for vision. Although many visual pigments have been documented for many fish species, a full overview of the genome content is limited to a few species, most of them fresh water teleosts. In the current study we have used the recently sequenced Atlantic cod genome [42] in order to investigate the visual pigment repertoire of Atlantic cod. To our knowledge this study provides the first molecular evidence for color vision in a teleost being driven by only two subfamilies of visual opsins. We have further used *in situ* hybridization to examine the expression pattern of different paralogs of each cone opsin subfamily in both adult and larvae. Our study shows that the genes belonging to UV and LWS subfamilies have been lost in Atlantic cod, while *SWS2* and *RH2* gene members have been retained in the genome. Furthermore we demonstrate that there have been duplications within both SWS2 and RH2 subfamilies in the cod ancestor, and also show that the expression pattern of opsin paralogs are different in the larva compared with the adult. We also show that the cone opsins are expressed at specific topographic locations within the retina of cod larvae.

## Material and Methods

### Synteny analysis and phylogeny of cone opsins in Atlantic cod

**Figure 1:**
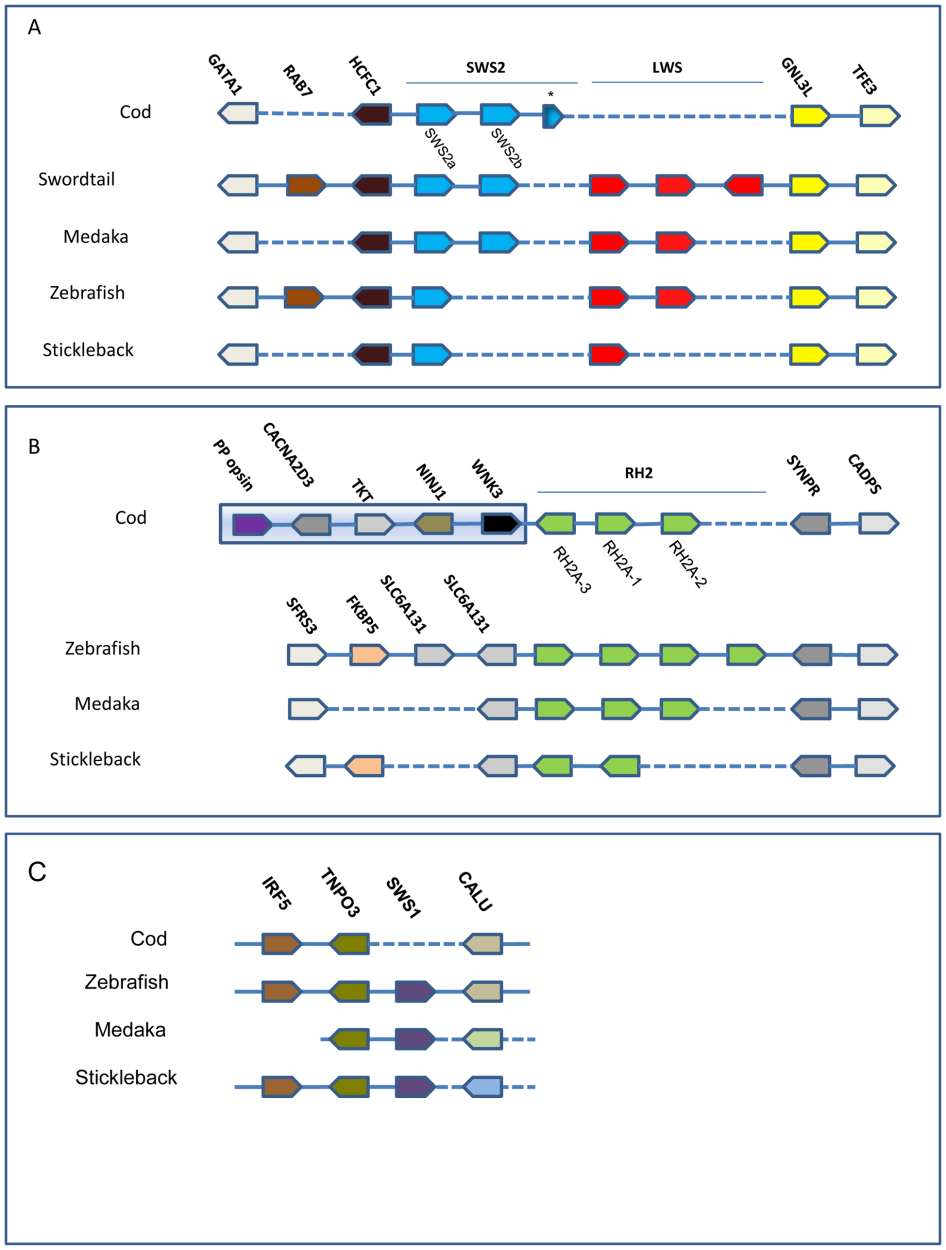
Synteny of cod cone opsins. (A) The scaffold ATLCOD1Cs2467 was found to contain the *SWS2-LWS* opsin syntenic region conserved within teleosts. The position of the *SWS2* genes and the phylogenetic analysis indicates that cod displays the same picture as seen in medaka with the *SWS2* genes being *SWS2A* and –*B*. The SWS* is an incomplete pseudogene. The genome lacks the *LWS* gene(s) and the region between the cod *SWS2B* and the *GNL3L* in cod is only 4.5 kb. (B) The scaffold ATLCOD1Cs169 was found to contain the *RH2A* opsin syntenic region. The number and orientation of the *RH2A* genes differs between the species, but the surrounding genes are the same except for cod which is linked to different genes upstream of the *RH2A* genes. The distance between the green opsins and the non-visual parapinopsin in cod is approximately 180 kb. (C) The scaffold ATLCOD1Bc1499404 was found to contain the *SWS1* opsin syntenic region. The closest genes surrounding the *SWS1* gene in zebrafish are also clustered in cod, but the *SWS1* gene is missing. The genes downstream of *SWS1* are different in medaka and stickleback.

**Figure 2:**
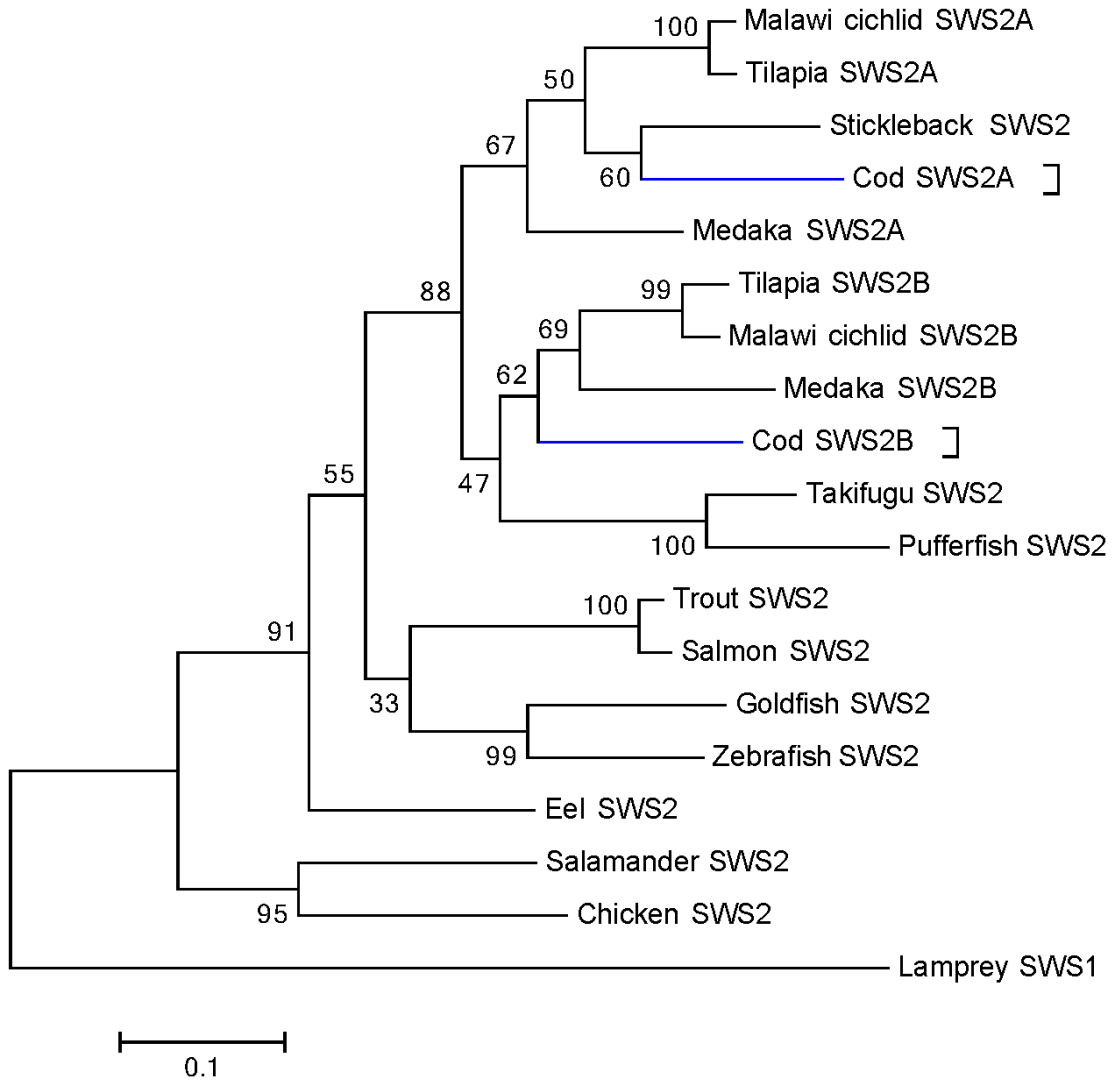
Phylogeny of blue-sensitive SWS2 opsins in teleosts. Deduced amino acid sequences were aligned with Clustal W, and the tree was generated by maximum likelihood method. The bootstrap confidence value (1000 replicates) is shown for each branch, and the lamprey (*G. australis*) SWS1 (accession number: AAR14684) sequence was used as outgroup. The scale bar is equal to 0.1 substitutions per site. Cod SWS2 genes are highlighted in blue. The protein sequences used for generating the tree are: Cod (*G. morhua*), Q5K6I6 and KJ572531 (translated); Malawi cichlid (*M. lateristriga*), Q4VPY3 and Q4VPX4; tilapia (*O. niloticus*), Q9I9I7 and Q9I9I9; salmon (*S. salar*), Q6XR07; trout (*O. mykiss*), Q7ZT59; Takifugu (*T. rupri*), Q6J5J9; zebrafish (*D. rario*), Q9W6A8; goldfish (*C. auratus*), P32310; guppy (*P. reticulata*), Q0H3C3; European eel (*A. anguilla*), ACT34385; chicken (*G. gallus*), NP_990848; tiger salamander (*A. tigrinum*), AAC96069 and spotted green pufferfish (*T. nigroviridis*), Q6J5J8.

**Figure 3:**
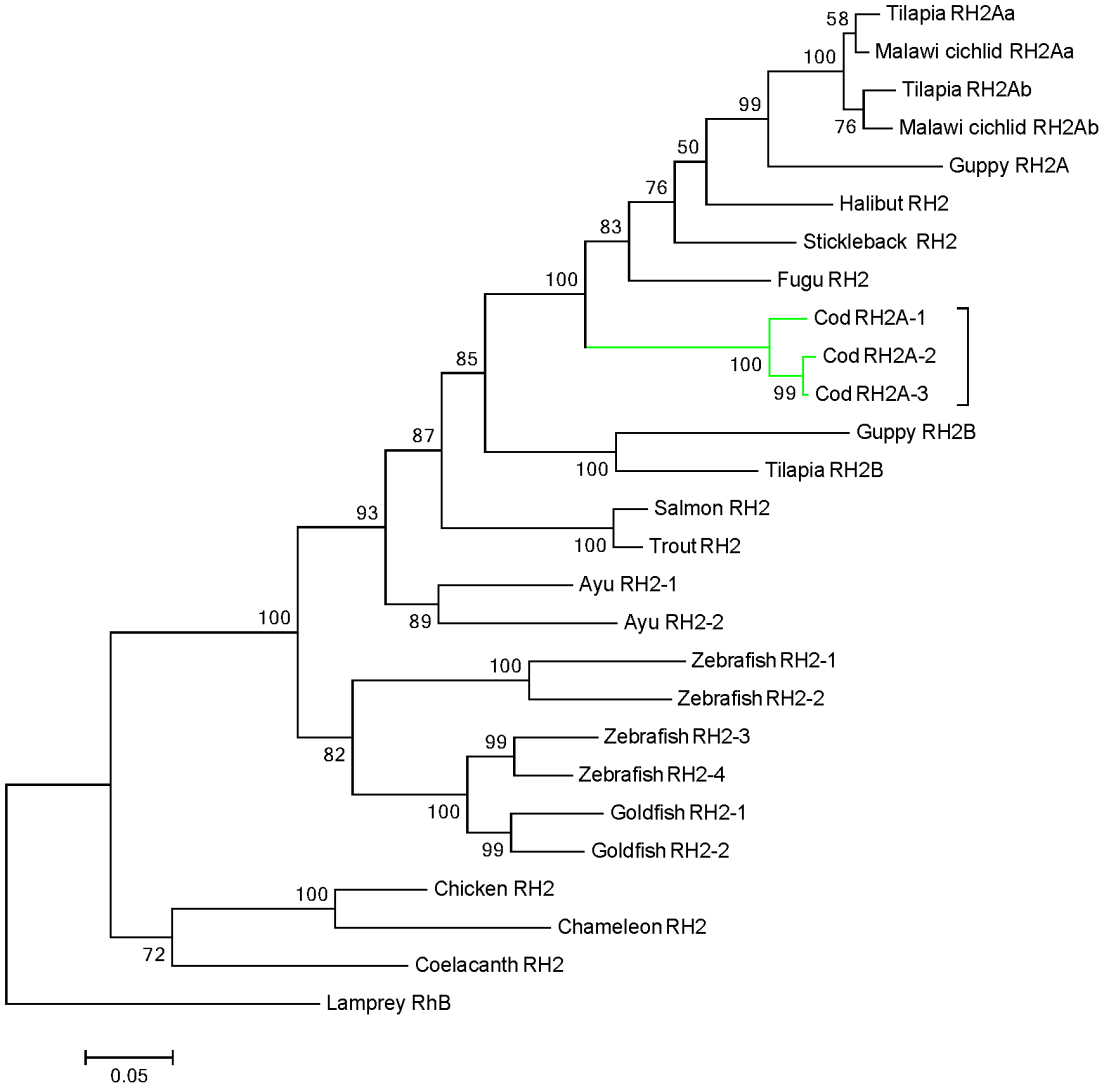
Phylogeny of green-sensitive RH2 opsins in teleosts. Coding nucleotide sequences were aligned with Clustal W, and the tree was generated by maximum likelihood method. The bootstrap confidence value (1000 replicates) is shown for each branch, and the lamprey (*G. australis*) sequence (accession number AY366494) was used as outgroup. The scale bar is equal to 0.05 substitutions per site. Cod RH2A genes are highlighted within the green box. The sequences used for generating the tree are: Atlantic cod (*G. morhua*), AF385824, KJ572530 and KJ572531; Malawi cichlid (*M. pyrsonotos*), ADI77346 and ADI72269; tilapia (O. niloticus), ADW80524, ADW80525 and ADW80526; guppy (*P. reticulata*), ABB69697 and ABB69696; halibut (*H. hippoglossus*), AAM17916; stickleback (*G. aculeatus*), AGL76515; fugu (*T. rupri*), AAF44648; salmon (*S. salar*), AAP58323, trout (*O. mykiss*), AAP35093; ayu (*P. altivelis*), BAD54744 and BAD54745; goldfish (*C. auratus*), AAA49168 and AAA49169; zebrafish (*D. rario*), AAD24752, AAD24753, BAC24131 and BAC24130; American chameleon (*A. carolinensis*), AH007735; chicken (*G. gallus*), NM_205490 and coelacanth (*L. chalumnae*), AAD30520.

TBLASTX was used to identify the opsin gene repertoire and the locations of them in the scaffolds of the reference genome (Fig. 1), using both the ATLCOD1C Newbler annotated version and the ATLCOD1B Celera assembly (http://www.codgenome.no/). For the genes where no cod sequences were available and for the other genes in the syntenic regions (see S1 and S2 Figs. for aa alignment), zebrafish and halibut sequences were used as query. Deduced aa sequences and coding nucleotide sequences were aligned with Clustal W [43], and the tree was generated by the maximum likelihood method using the Jones-Taylor-Thornton (JTT) substitution model for amino acids (SWS2) [44], and the Tamura-Nei model for nucleotide substitution for RH2 coding sequences [45] with bootstrap confidence of 1000 replicates and uniform rates among sites. Both models are implemented in the MEGA 5.0 software [46]. For the construction of the SWS2 tree, lamprey (*G. australis*) SWS1 was used as outgroup, and for the RH2 tree; lamprey RhB was used as outgroup. For further information concerning sequence accession numbers; see figure legend for Fig. 2 (SWS2) and Figure 3 (RH2).

### Biological material

Newly fertilized coastal Atlantic cod (*Gadus morhua*) eggs from one female fish were donated by Austevoll Aquaculture Research Station, near Bergen, Norway, which has permits to both catch and maintain all stages of Atlantic cod, including the parent fish of the donated fish eggs. The fertilized eggs were allowed to develop to 18 days post fertilization (dpf) (corresponding to 3 days post hatching) in 0.5 L containers with oxygenated sea water at approximately 6°C at a facility located in Bergen Hightechnology centre (HiB) (Norwegian Animal Research Authority approved; code 018). The larvae were sampled at 12.00 p.m. (6 hours after light was turned on) and euthanized with an overdose of methacaine (MS-222, Sigma, USA) and when movement of larvae was no longer detected, the larvae were transfered into icecold fixative (paraformaldehyde), see section 2.3 and 26 for further details. The light regime was 12∶12 hour light-dark. The cod used for studies on adult life stages was caught in its natural environment on the west coast of Askøy at approximately 11.00 p.m. (GPS coordinates: Latitude 60.478521, longitude. 5.003244). The weight of the juvenile cod presented in the current study was 165 g measuring 26 cm. The use and handling of animals in the current study was performed aaccording to Norwegian law and the Norwegian Animal Research Authority (NARA) following procedures at the authorized facility. According to NARA the use of fish larvae at stages prior to first feeding is not recognized as research animals as long as the involved procedures do not affect larvae at later life stages, hence the current study do not demand a legal approval from the local Animal Care and Use Committee (IACUC).

The juvenile/adult fish used in the current study was caught in the wild by the authors of this paper by the use of a fishing rod and immediately euthanized by a blow to the head, then bled out by cutting the main artery. The fish was caught in an area and by methods approved by the Norwegian Directory of Fisheries and by the local ethical committee for animal care and use (see section 2.6. *Ethics Statement* for further details). As the sampling of tissue was done after the fish was dead, the fish was not considered as being a research animal according to the Norwegian law of Animals in Research, hence further analysis on tissues obtained from this animal did not require any additional approval. Atlantic cod is not considered being an endangered species in Norway.

### Tissue preparation for in situ hybridization

Cod larvae were sampled on liquid nitrogen for RNA isolation and fixated in ice cold 4% paraformaldehyde (Sigma, USA) in 1x phosphate-buffered saline (PBS) solution (pH 7.4) for in situ hybridization. The adult cod were euthanized by a blow to the head and decapitation, and the eyes immediately dissected out and lens removed. The dissected eyes were fixated in 4% paraformaldehyde in 1x PBS for sectional *in situ* hybridization. Both larvae and dissected eyes were fixed for 48 hours then transferred to 20% sucrose in PBS and left overnight. For retinal flat mount the retina was carefully dissected out of the sclera and transferred to glass slides. Both eyes and larvae were imbedded in a gradient of 20% sucrose-TissueTech (Chemiteknikk, Norway) and 100% TissueTech on an icecold metal block for rapid solidification. Frozen tissues were cut with a Leica CM3050S cryostat (Leica, Germany) into sections of 10 µm.

### Molecular identification of opsins

Extensive searches of visual opsins by our lab using degenerated primers identified opsins belonging to only two subfamilies of cone opsins; the SWS2 and RH2. The use of degenerated primers in screening for visual opsins in halibut, have previously been described by our lab [29], and the same procedure and primers were used on cod. No opsin genes of SWS1 or LWS subfamilies were found. Further *in silico* searches in the available cod genome database confirmed these findings and further identified an additional blue SWS2 opsin and two green RH2 opsins. A total of one blue SWS2 and two green RH2 cone opsins were cloned in the current study. The genes have been assigned the following GenBank accession numbers: *RH2A-2*: KJ572530, *RH2A-3*: KJ572531 and *SWS2b*: KJ572532. A second SWS2 and a third RH2 gene (RH2A-1) has previously been cloned (GenBank: AF385822 and AF385824, respectively).

Total RNA was extracted from several developmental stages according to the method which has been described [47]. The RNA was then DNase treated with RQ1 RNase-Free DNase (Promega, USA), and cDNA synthesized using reagents from Promega with oligoDT as primer according to manufacturer's guidelines. The cDNA was pooled from several developmental stages and primer pairs for each opsin (S1 Table) were used in a PCR to generate specific bands. The bands were cut from a 1% agarose gel, ligated into the Strataclone vector (Agilent, USA), RH2A opsins and pGEMteasy vector (Promega), SWS2 opsins, and cloned into competent *E.coli* (Invitrogen, USA). To extract plasmids, a midiprep (Qiagen, Germany) was performed on positive colonies following manufacturer's instructions. All positive plasmids were sequenced using the 3730XL Analyzer (Applied Biosystems, USA) at the University of Bergen Sequencing Facility (Norway). The obtained sequences of available cone opsins were confirmed by searching the sequences against the annotated Cod genome database (NCBI, USA) and GenBank (NCBI) against other species, in both cases using BLASTN and TBLASTX algorithms.

#### Real time (RT)-PCR

A RT-PCR was performed (S3 Fig.) to determine which developmental stages the various cone opsins where present, and allowed for selection of stages used in *in situ* hybridization. The RT reaction was performed on the same material and cDNA as previously described, however without pooling the stages. Three developmental stages were included; 12 dpf, 18 dpf and adult (total RNA isolated from whole retina, otherwise cDNA synthesis similar as previously described). The primers used in the RT-PCR reaction was the same as used for probe synthesis (see section 2.5.). The EF1a was used as an internal reference gene and has previously been described as a suitable candidate in cod [48] The primers used for EF1a was; EF1a_fw1: CCACCGGCCACTTGAT and EF1aRv1: GCTCTGCCAAGGTCACCAAG, which produced a band of 1292 bp. The RT-PCR was performed using the Advantage 2 PCR Enzyme system and settings (Clontech, USA), however with the number of cycles and the primer annealing temperatures (Ta) optimized (according to primer melting temperature) for the respective genes: EF1a; 25 cycles; Ta 66°C, SWS2A: 35 cycles; Ta 64°C, SWS2B: 35 cycles; Ta 67°C, RH2A-1: 35 cycles; Ta 59.5°C, RH2A-2: 35 cycles; Ta 63.5°C and RH2A-3: 35 cycles; Ta 61.5°C.

### In situ RNA probe synthesis and hybridization

In the present study RNA probes for *in situ* hybridization studies were designed based on a combination of the available sequence information of the cloned opsins and searches in the cod genome database. For the RH2 opsins (*RH2A-1*, *RH2A-2* and *RH2A-3*) probes were designed in the 3′UTR region of each transcript in order to avoid cross-hybridization of probes in the highly conserved coding sequence (CDS). For RH2 nucleotide alignment and location of area used for probe synthesis; see S4 Fig. The SWS2 opsins showed less conservation in the CDS region (79% nucleotide similarity), and these probes were therefore designed to cover a largest possible area of the transcript to maximize the sensitivity. DIG labeled probes were designed and prepared according to the method described for zebrafish [49]. Antisense probes were designed by including the sequence of the T7 polymerase binding site (5′-TAATACGACTCACTATAGGG-3′) in the reverse primer for each transcript, while sense probes were designed by including the T3 polymerase binding sequence (5′-CATTAACCCTCACTAAAGGGAA-3′) in the forward primer, see S1 Table for primer specifications (T7 and T3 binding sequences are not shown in S1 Table). Both T7 and T3 sequences were included in the 5′ extremity of each primer.

*In situ* hybridization on sections was performed according to the method previously described [28]. For whole mount *in situ* on cod larvae, fixated larvae were briefly washed in 1xPBS, then dehydrated in methanol and stored at −20°C in 100% methanol until use. The procedure was initiated by rehydrating the larvae in methanol (75–25%) following a wash for 2×5 minutes in 1xPBS pH 7.4. The larvae were bleached with 3% H_2_O_2_/0.5% KOH (Sigma) in order to remove eye pigmentation. The bleaching reaction was stopped with 1xPBS for 5 minutes, then 4×5minutes in 1xPBSTw (0.1% Tween20 (Sigma), in 1xPBS). The tissue was opened with proteinase K (Promega) treatment (10 µl/ml in 0.1 M Tris-HCl pH 8.0 and 50 mM EDTA). After a washing step with 1xPBSTw the larvae were fixated in 4% paraformaldehyde-buffred PBS (pH 7.4), then washed 4×5minutes in 1xPBSTw. Prehybridisation was carried out at 65°C for 2 hours in hybridisation solution without probe prior to incubation with hybridisation solution with probe for 16 hours at 65°C. The hybridisation solution was prepared with 10 mM Tris-HCl pH 7.5, 300 mM NaCl, 1mM EDTA, 0.2% Tween20, 1% Blocking reagent (Roche Diagnostics, Germany), 10% dextransulphate (Sigma) and 50% formamide (VWR, USA). After hybridisation a washing series of 2×15 minutes in 50% formamide (VWR) in 2xSSCTw (sodium-sodium citrate (Sigma) buffer with 0.1% Tween), 1×30 minutes in 2xSSCTw, 2×15 minutes in 2xSSCTw and 2×15 minutes in 0.2xSSCTw was performed at 65°C. To remove unhybridised probe the larvae were treated with RNase A (0.02 mg/ml) (Sigma) for 20 minutes at 37°C before washing with RNase buffer (10 mM Tris-HCl pH 7.5, 0,5 M NaCl, 1 mM EDTA) for 20 minutes at 65°C. The embryos and larvae were incubated in 2xSSC with 0.05% TritonX-100 (Sigma) and 2% Blocking reagent (Roche Diagnostics) for 2–3 hours before overnight incubation with Anti-Digoxigenin-Alkaline phosphatase, Fab fragments (1∶2000) (Roche Diagnostics) in 2xSSC, 1% Blocking reagent (Roche Diagnostics) and 0.3% TritonX-100 (Sigma). The larvae were first washed for 4×20 minutes in 1xPBSTw to remove redundant antibody, then for 2×10minutes in visualisation buffer (100 mM Tris-HCl pH 9,5, 100 mM NaCl, 50 mM MgCl_2_). The staining reaction was performed in darkness with freshly made chromogen substrate (45 µl 4-Nitro blue tetrazolium chloride and 35 µl 5-Bromo-4-chloro-3-indolyl-phosphate (Roche Diagnostics) in 10 ml visualisation buffer. All probes were tested in parallel with a sense probe as a control of unspecific binding. No signal was observed for any of the sense probes. The staining was terminated by washing the larvae in stop solution (10 mM Tris-HCl pH 7.5, 1 mM EDTA and 150 mM NaCl) before mounting in 100% glycerol (Sigma). Sections were mounted in 70% glycerol (Sigma) in 1xPBS. Pictures were taken with a Leica 6000B microscope for sections and a Leica M420 for whole larvae.

### Ethics Statement

The Austevoll Aquaculture Research station has the following permission for catch and maintenance of Atlantic cod: H-AV 77, H-AV 78 og H-AV 79. These are permits given by the Norwegian Directorate of Fisheries. The Austevoll Aquaculture Research station has furthermore permit to run as a Research Animal facility using fish (all developmental stages), with code 93 from the national IACUC; NARA. Although the sampling of stages of larval cod used in the current study do not require a specific permit according to Norwegian law of Animals in Research, Regulation of the 15^th^ of January 1996; the method of euthanization of fish larvae with the use of methacaine follows § 16 Euthanasia of Laboratory Animals stating that the choice of euthanasia should involve no signs of unnecessary suffering of the animal. Methacaine has been shown to be an acceptable and efficient euthanasia in fish [50].

Adult cod were caught by the authors. Atlantic cod are not considered endangered or vulnerable. No permits were required to catch wild Atlantic cod as long as one uses a standard fishing rod (http://lovdata.no/dokument/SF/forskrift/2006-10-13-1157), as was the case in the current study. However the University of Bergen, Institute of Biology where the person in charge of the sacrification of cod is an employee, has the permit to catch Atlantic cod in all developmental stages/sizes given by the Norwegian Directory of Fisheries (reference number: 12/14048, dated 05.06.2013. This permit covers the region on the west coast of Bergen where the wild Atlantic cod was caught, approximate GPS coordinates: Latitude 60.478521, Longitude 5.003244.

## Results

### Identification of cone opsins in cod

An approach combining the use of degenerated primers (prior to sequencing of the cod genome) and *in silico* searches in the cod genome were used in the identification of a total of five cone opsin genes belonging to only two cone opsin subfamilies; the blue sensitive SWS2 and the green sensitive RH2 (Fig. 1A, 1B). While the *RH2A-1* (previously called *RH2-1*) and *SWS2A* gene coding sequences was previously reported in GenBank (NCBI), we cloned two additional RH2 genes; *RH2A-2* and *RH2A-3* and one additional SWS2; *SWS2B*. Full-length cDNA sequence was obtained for *RH2A-1* and *SWSB*, while partial sequences were obtained for the remaining genes. However in all cases enough sequence was obtained to confirm that the cloned genes corresponded to the opsin sequences available in the cod genomic ensemble library and to annotate cone opsins in various species through blast search (GenBank). All of the deduced protein sequences showed typical opsin characteristics, including seven transmembrane domains and conserved residues involved in chromophore binding pocket (S1, S2 Figs.). We did not find any cone opsin members of the SWS1 or LWS families. By comparing the syntenic region containing *SWS1* in cod with other teleosts, we find that although these genes are missing in cod, the genomic region both upstream and downstream is otherwise maintained (Fig. 1C).

The *SWS2A* and *SWS2B* encoded 351-, and 352 -amino acids (aa), respectively (showed 75% similarity). When generating a phylogenetic tree with cod opsin sequences, we found that the two blue was placed within two separate main clusters where cod SWS2A was grouped with medaka, tilapia and cichlid SWS2A, while cod SWS2B was grouped together with stickleback, medaka, tilapia and cichlid SWS2B (Fig. 2). The synteny analysis showed that the two SWS2 genes were located in a common chromosomal region (tandem linked) (Fig. 1, A), only 930 bp separating the coding sequences of each gene (not shown). Furthermore, in addition to the two functional intact *SWS2* genes we also identified remnants of a third nonfunctional SWS2 gene adjacent to the *SWS2B* in cod (Fig. 1A), with deduced aa sequence coding 109 aa corresponding to the third-to-fifth transmembrane regions of the *SWS2* genes. The SWS2 containing chromosomal region and the entire cod genome were shown to be completely void of any LWS genes (Fig. 1A). The current finding is in contrast to what is shown in other teleosts (Fig. 1A).

Three green opsins were identified in which the RH2A-1 mRNA sequence has previously been published on NCBI (Accession number: AF385824.1). Protein sequences were predicted for all RH2- genes based on the respective transcript, and encoded 347 aa for RH2A-1, 349 aa for RH2A-2 and 356 aa for RH2A-3. The RH2 encoding genes were found to be highly conserved (RH2A-2 and RH2A-3 show 99% aa similarity, and RH2A-2 and RH2A-3 show 94% and 96% similarity, respectively, with RH2A-1), and only differed from each other at a few aa positions (S2 Fig.). The phylogenetic analysis of green opsin coding sequences showed that all three RH2 genes were clustered together within one main branch; however indicates that RH2A-2 and RH2A-3 may be slightly more similar to each other, than to RH2A-1 (Fig. 3). The cod RH2 opsins were again clustered together with the RH2A opsins of cichlids and tilapia, together with RH2A opsins of fugu, stickleback and halibut (Fig. 3).

Similar to other species, the three RH2A genes were found to be linked in tandem within a region of approximately 15 kb (Fig. 1B). While the downstream region of the cod RH2A cluster follows the synteny from other teleosts, the upstream region differs in cod compared with other fishes included. A genome rearrangement has placed the RH2A genes in the vicinity of another opsin gene, a parapinopsin. In contrast, both the RH2 upstream and downstream syntenic region is conserved in other teleosts. As for the SWS2 and LWS genes, the genomic region surrounding RH2 genes is conserved among several fish species.

Our RT-PCR on selected developmental stages (S3 Fig.) confirms that all cone opsins are present and expressed in cod. Our survey suggests that opsin expression is initiated after 12 dpf, and all available cod cone opsins are expressed in the first-feeding 18 dpf cod larvae. The adult however seems to have lost expression of RH2A-2 and RH2A-3, or undetectable levels (see section 3.2, for variation in opsin expression). Our gel analysis further shows that there is only a single band present for the three highly conserved RH2A paralogs.

### Ontogenetic changes in the array of visual pigments

**Figure 4:**
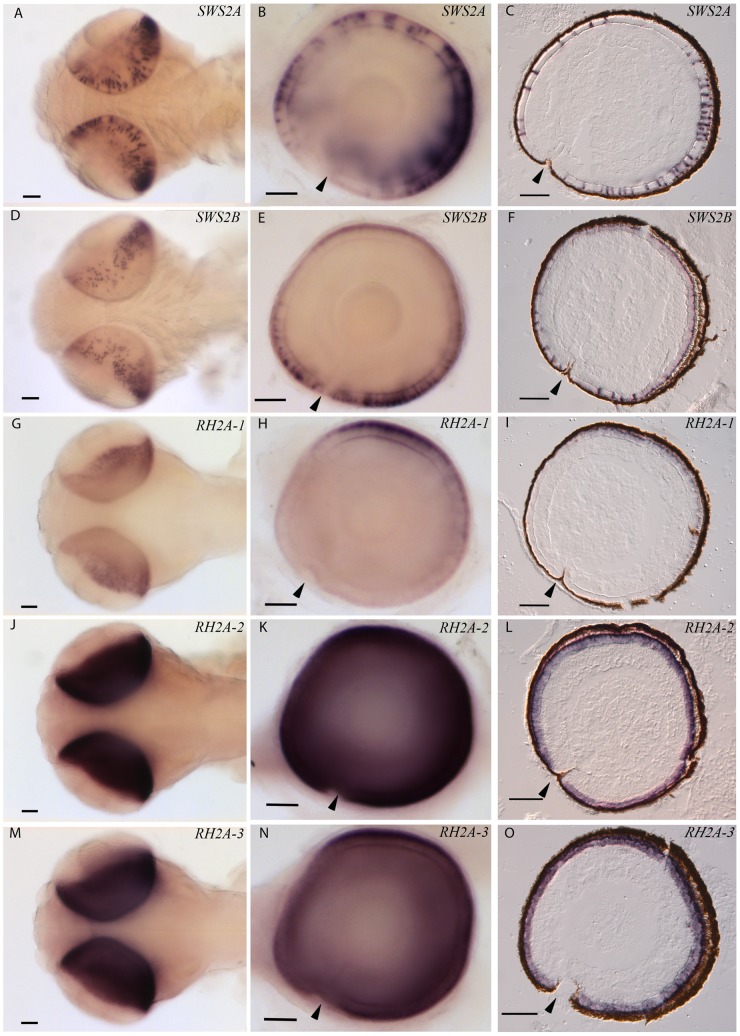
Topographic mRNA expression of visual opsins in cod larvae. The retinal expression of different cone opsins were investigated by *in situ* hybridization on 18 days post fertilized cod larvae (2 days post hatching). The pictures in the left row (A, D, G, J, and M) show dorsal view of whole mount in situ on larvae, while the centre row of pictures (B, E, H, K and N) show whole mount of a right eye, lateral view; with larval anterior to the left. Sectional in situ is shown by sagittal sections in the left row of pictures (C, F, I, L and O). Expression of the various cone opsins was visualized by specific dig-labelled RNA probes: *SWS2A* (A, B and C), *SWS2B* (D, E and F), *RH2A-1* (G, H and I), *RH2A-2* (J, K and L) and *RH2A-3* (M, N and O). Arrows indicate location of choroid fissure. Scale bars, 500 µm.

In order to characterize color vision in cod, we analysed the expression of the five cone opsins by the use of *in situ* hybridization technique using gene specific DIG-labelled RNA probes. To be able to distinguish expression of the highly similar RH2A genes, probes were designed in the 3′UTR regions. To examine potential ontogenetic differences, both larval and adult stages were included in the study. Both sectional- and whole-mount *in situ* hybridization on 18 days post fertilized cod larva (2 days post hatching) show that all five cone opsins are expressed in the retina (Fig. 4, A–O). The two blue-SWS2 genes were both expressed in a higher number of cone cells in the ventro-temporal region of the retina (Fig. 4, A–F). The *SWS2A* mRNA seemed to be located in a larger part of the cone cytoplasm, or in larger cone cells compared with the cones expressing *SWS2B* and all of the *RH2A* genes (Fig. 4, A–C). Both SWS2A and SWS2B expressing cone cells were found at some distance apart.

The cod larval retina was found to be dominated by expression of the RH2A-2 and RH2A-3 subtypes, both of which are expressed throughout retina (Fig. 4, J–O). In contrast, the RH2A-1 expressing cones were most abundant in the dorso-temporal retina (Fig. 4, G–I). Each of the three green-sensitive RH2A genes were found to be expressed in a dense pattern and the mRNA seemed to be most abundant in the proximity of the photoreceptor outer segment, compared with the more easily distinguishable SWS2 expressing cones.

**Figure 5:**
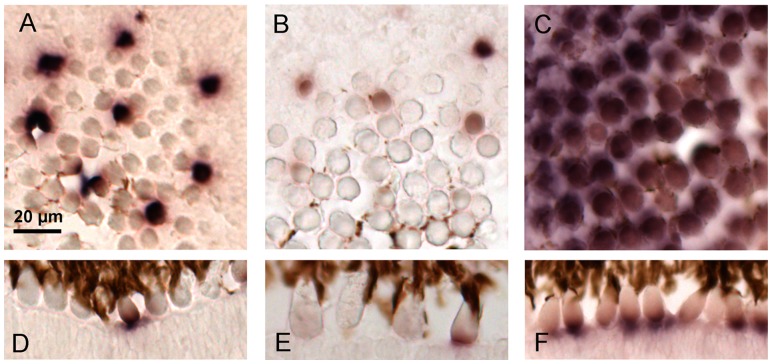
Cone opsins expressed in adult cod. *In situ* hybridization shows that adult cod express both subtypes of *SWS2*; *SWS2A* and *SWS2B*, while only one RH2A subtype, the *RH2A-1* is expressed in the retina. The *SWS2A* opsin (A and D) seems to be expressed in a higher number of transcripts in each photoreceptor cell compared with the *SWS2B* opsin (B and E). Both *SWS2A* and *SWS2B* expressing photoreceptors are found at some distance apart, and appear to form a regular pattern. The green RH2A-1 opsin (C and F) dominates in the retina of adult cod with only a few cells lacking expression. Scale bar, 20 µm.

In adult cod we found a change in the profile of expressed visual pigments. *In situ* hybridization on cryosections of adult cod retina detected expression of both SWS2 genes (Fig. 5, A, B, D, E), which is similar to the situation in cod larva. However in contrast to the larva, only one of the three RH2A genes (*RH2A-1*) was expressed in photoreceptor cells of adult cod (Fig. 5, C, F). The gene expression data showed that the green *RH2A-1* expressing cones dominate in the adult retina with only a few cells lacking expression (Fig. 5, C, F). In contrast, the cones expressing the two SWS2 opsins were more scarcely spread (Fig. 5, A, B, D, E). The *SWS2A* gene seemed to be higher expressed in each photoreceptor cell, compared with the *SWS2B* gene, however probe efficiency could influence the strength of the signal and the data should thus be interpreted in a qualitative manner (Fig. 5, A, B).

## Discussion

### Genome organization of cone opsins and adaption to the marine environment

In the current study we show that the cone opsin repertoire of the Atlantic cod genome is restricted to only two subfamilies of visual pigments; SWS2 and RH2, sensitive in the blue and green part of the spectrum. We further show that the three members of the RH2 subfamily are differentially expressed in cod larvae and juveniles, while the two members of the SWS2 family are expressed in both of these developmental stages.

Analysis of the cod genome verifies the existence of only two cone opsin gene families, SWS2 and RH2 (RH2A). These genes are located in conserved regions with synteny among teleosts. Examination of the genomic regions where the missing opsin subfamilies, SWS1 and LWS genes, are located in other teleosts, confirms a loss of these genes in cod. Since both upstream and downstream genes in these regions are still intact, our finding indicates a loss of these opsin genes through evolution, possibly through accumulation of mutations. To our knowledge this is the first report describing the loss of visual pigment gene families known to be sensitive in the shortest and longest part of the visual spectrum. Loss of whole opsin gene families are also known from mammalian evolution where in contrast to cod the two cone opsin families with light detection in the central part of the light spectrum (SWS2 and RH2) were lost in the “Nocturnal period” [51]. In the human linkage, spectral sensitivity in the central region of the light spectrum was improved by gene duplication of the LWS gene that originated in primates [4], [52]. In principle opsin evolution in cod seems to have followed a similar pattern where both loss of whole gene families and gene duplications have shaped the current visual system.

Earlier MSP studies on adult cod only detected two types of photoreceptors sensitive in the blue and green region, respectively [20], which is in accordance with our findings. Since we lack in vitro expression and spectral analysis on all paralogs of cod visual pigments (including those only present in cod larvae), we cannot conclude that cod colour vision is non-functional within the UV or the red part of the light spectra. However, structurally we find it unlikely that any of the SWS2 paralogs are tuned up to 100 nm, shifting peak absorbance from blue to UV. Even though it has been shown several times in ray-finned fishes that convergent evolution at RH2 opsin key sites may result in a blue shift, such tuning towards the red part of the spectra have not been described [11]. Furthermore, we find that there is a lack of amino acid substitution in RH2 tuning sites in the highly conserved cod RH2A paralogs, indicating that none of the paralogs are tuned into the red area of the light spectra (neofunctionalized) and taken over the role of LWS opsins. This study of Atlantic cod shows that loss of opsin subfamilies sensitive towards UV and red light has been part of the evolution of cod color vision. Whether the loss of UV and LWS opsins is a specific feature in Atlantic cod or is a common feature for the whole Gadiformes branch, needs to be further verified.

### Tandem duplication and phylogenetic evolution

Both SWS2 and RH2A genes are clustered in conserved blocks of the chromosome, with the exception of the upstream region of RH2A (Fig. 1, A, B). A genomic organization of visual pigments within the same cone opsin family in tandem clusters has been reported for zebrafish [41], swordtail, *Xiphophorus helleri* [53], guppy, *Poecilia reticulata* [54], medaka, *Oryzias latipes* [40] and cichlids [55], suggesting that this is a common feature conserved among fishes. A recent study on opsin duplication and evolution in ray-finned fishes suggest that opsin genes have duplicated multiple times spanning the evolution of this group, and interestingly this is suggested to have occurred mainly through tandem duplications [11]. The authors suggest that tandem duplication is the most common form of opsin duplication in fishes, and that potential consequences may be co-regulation of opsins [11], [56]. To elucidate whether opsins are co-regulated in cod, more studies are needed.

The conservation of a physical chromosomal linkage of SWS2 and LWS visual pigments is considered to represent the ancestral gene arrangement, and has been described for species in all vertebrate classes [57], [58]. Although cod has lost the LWS genes, both downstream and upstream genomic regions are conserved (Fig. 1, A). Our phylogenetic analysis indicates that the cod SWS2A and SWS2B opsins are clustered together with the SWS2A and SWS2B of other species (Fig. 2). The branching event causing the divergence of SWS2A and SWS2B groups has previously been described to have originated in the Acanthopterygii early ancestor groups, or in the ancestor of the clade Holacanthoptergyii including the Acanthopterygii group and the Paracanthoptergyii group that includes cod [10], [11]. The duplication event producing the SWS2A and SWS2B groups have been shown to be one of two duplication events in the SWS2 opsins, the second duplication taking place in a cyprinid ancestor [11]. Although we do not have in vitro spectral data on the two SWS2 genes, the SWS2A and SWS2B groups have been shown to be spectrally different in medaka, cichlids and tilapia [40], [59], [60]. We also detected remnants of a third SWS2 member (pseudogene) within the same SWS2-cluster, suggesting that there may have been more than two SWS2 members in the cod ancestor. Whether the two cod SWS2 genes are spectrally different remains to be shown, however none of the amino acids differ in the chromophore binding pocket [39].

The three tandem repeated RH2A genes of cod are clustered together in one phylogenetic branch as a result of high sequence similarity of RH2A subtypes (Fig. 3). In contrast to the SWS2 subfamily, duplication events within the RH2 opsins have occurred several times through evolution, and in tilapia (*O. niloticus*) several tandem duplications within the same locus have produced three linked RH2 genes (RH2B, RH2A and RH2Aa) [11]. While a more ancient duplication event produced the RH2A and RH2B clades, a more recent tandem duplication in the intralacustrine ciclid radiation have resulted in the RH2Aa and RH2Ab groups [10], [11]. Similar to these findings our analysis suggests that the RH2 genes found in cod are a result of multiple duplications through evolution. All cod RH2 opsins are placed within the RH2A group (Fig. 3), suggesting that the RH2A paralog have been duplicated at least at two occasions after the split of the RH2A and RH2B clades. These data further suggest that the more ancient RH2B paralog most likely have been lost in evolution of the cod lineage. Independent duplication of both RH2A genes and RH2B genes have been reported in a number of species (RH2A; stickleback *(G. auculeatus*), seabream (genus: *Acanthopagrus*), medaka (*O. latipes*) and cichlids, and RH2B; lanternfish (S. leucopsarus)), that together with our findings support the hypothesis that this is a common feature within the RH2 subfamily [11]. The presence of three highly similar RH2A paralogs suggests that these recently have been tandem duplicated. Still, this picture is unclear and the role of gene conversion in reducing divergence between duplicated gene copies is uncertain. However it should be noted that gene conversion is facilitated by tandem duplicated paralogs. Thus we cannot rule out that some or all of the cod RH2A paralogs have reduced genetic variation due to over-writing by a functional ancestral “donor” gene [11]. Although we do not know the maximum spectral absorbance of each RH2A subtype, or whether this may differ among subtypes, our analysis of the cod RH2A alignment did not detect any amino acid substitutions in known tuning positions [39]. It is thus unlikely that the three RH2A subtypes are tuned significantly different. On the other hand, the presence of three RH2A encoding genes may provide more transcripts, that when translated could allow improved absorbance of light in the area of maximum absorbance. This is however speculative at the time, and more studies are needed to test such a hypothesis.

### Adaption to the blue-green photic environment

Despite the wide range of spectrally different cone opsins in teleosts, the number of cone classes present in each species may vary according to both ontogeny and changes in the photic environment. In our study we show that cod lacks the genes coding for visual opsins sensitive in the UV and red field of the light spectra. Although both the coastal and continental shelf environment which cod inhabits is dominated by blue-green light, light in the ocean rapidly changes and UV and red light is present in the upper levels of the water column where cod larvae is found, which may favour retention of opsins with various sensitivities. However, despite the environmental heterogeneity in the ocean, cod has lost the UV and LWS opsins, suggesting that there isn′t always a clear relationship between present opsins and habitat, thus loss of these subfamilies could be due to gene loss in the cod ancestor, rather than a direct adaptive mechanism in cod (see Rennison *et al*. 2012 [11], for further discussion). In contrast, the array of SWS2 and RH2A paralogs arising from gene duplications have been retained, and have enabled visual adaptation to the photic environment in such a way that cod is able to detect a prey and avoid a potential predator. Somewhat similar to cod, studies on eel show that the epipelagic freshwater yellow eel also possess RH2 and SWS2 cones, however as the freshwater eel goes through metamorphosis and becomes a deep sea silver eel, the cones are almost lost and the vision becomes rod based in the new scotopic conditions [33]. Atlantic halibut on the other hand, expresses all four cone classes sensitive to UV, blue, green and red, which in this case may be an adaption to a wide range of habitats ranging from the upper 20–60 m of depth during the first 4–6 years, and later to both shallow and deeper waters [28], [29]. A recent study in rainbow trout suggests that UV cones are important for zooplankton foraging in juvenile fish, however as the fish grows and changes prey type blue cones become more favourable [35]. In contrast to the epipelagic and pelagic zones, light conditions in the deep sea are more stable, and it may be more common to observe species with fewer subfamilies of opsins and even fewer paralogs within each subfamily, given that opsin duplication and divergence is driven by environmental heterogeneity [11]. Furthermore, one should also consider variations in opsin expression patterns, which have been shown to vary in a number of species including eel and rainbow trout previously mentioned.

### Visual pigments and ontogeny

Although the range of expressed opsins has been described for many species, less is known concerning ontogenetic usage of differential cone opsin subtypes, and even less is known about how this may affect behaviour. Although cod is not a deep sea species, the settlement of early juveniles into deeper waters most often involves a change in light conditions. We find the subsurface dwelling cod larvae to express all three green subtypes, while the adult cod seems to have lost expression of two RH2A subtypes and only expresses RH2A-1. However, the two blue opsin genes are expressed in both larvae and adults. Altogether these findings suggest life-stage specific genetic programs of opsin regulation. Previous MSP analysis of adult cod photoreceptors showed that single and double cones have wavelengths of maximum absorbance (λ_max_) at 446nm and 517nm, respectively [20]. These data suggest that adult cod RH2A-1 are most likely expressed in identical double cones which absorb maximally at 517 nm, however not distinguishing between the SWS2 subtypes. This could either indicate that both SWS2 pigments have similar spectral profile or that just one of the SWS2 cone populations was analysed by the random picking of photoreceptor cells for MSP analysis. To date, no MSP analysis has been conducted on cod larvae, so it remains to be shown whether the different RH2A subtypes are spectrally different. In vitro expression and spectral analysis of the different cone opsin paralogs are needed to confirm potential differences in sensitivities, and may be an easier approach than MSP since some of the opsin classes has low abundance in retina.

Ontogenetic plasticity in expressed cone opsins is a common feature in several species. The loss of UV corner cones in adult teleosts has been reported in a number of species [61]–[63]. Atlantic halibut however retain expression of both UV and red together with blue and green sensitive cones, though changes the relative expression of each cone opsin [28], [29]. Comparable to Atlantic halibut, salmonid fishes also express all four classes, which typically hatch with UV, green and red sensitive cones, however switches from UV to blue in the large juvenile rainbow trout while maintaining expression of green and red [35].

### Opsin expression topography in retina

In the current study we show that all available cod cone opsins are expressed in the larval retina by the use of both whole mount and sectional *in situ* hybridization. The topography of cone opsins we observe represents the larval retina present at the time prior to first feeding. At this stage the retina is dominated by green-sensitive cones, where RH2A-2 expressing cones are found in a dense pattern throughout retina (Fig. 4, J, K, L). A similar expression pattern is seen in RH2A-3 expressing cones (Fig. 4, M, N, O), together suggesting that these two subtypes show minimal or no topographic variance. The RH2A-1 subtype on the other hand, is expressed in a higher number of cones in the dorso-temporal region of the retina (Fig. 4, G, H, I). Similar to cod, topographic studies on halibut larvae also show that green-sensitive cones dominate and are spread evenly throughout retina [28]. In zebrafish the green sensitive cones are most abundant, and the topography of the four RH2 subtypes changes differentially both spatial and temporal [32]. The dominance of RH2 cones is also described in larvae of Winter flounder [64] and a number of northwest Pacific marine fishes [65], altogether suggesting that this is common in several teleost species. Given that green light is abundant in the epipelagic oceanic zone and in freshwater, a high abundance of green sensitive cones most likely increases the resolving power of the larval retina in this part of the light spectra. Our serial sectioning through an adult cod eye did not show any topographic variation in RH2A-1 expressing cones, and no remnants of RH2A-2 and RH2A-3 cones was found in any of the retinal regions, suggesting that these cones either is lost by apoptosis or have switched the expression into RH2A-1, SWS2A or SWS2B. More studies are needed to understand how this regulation is achieved.

The cod larval SWS2 subtypes are expressed in a less number of cones appearing at some distance apart (Fig. 4, A–F), a pattern which is also to some degree seen in larval halibut [28]. The SWS2A expressing cones are slightly more abundant in the temporal-ventral retina, suggesting that this part of the retina has greater resolving power in the blue spectra of the light field (Fig. 4, A–C). In situ hybridization on larval sections further show that while the SWS2a is expressed in a larger part of the cones, the SWS2B is expressed in a more flattened pattern in the cones located in the dorso-temporal region (Fig. 4, C, F). However, the SWS2A expressing cones in the naso-ventral retina show expression in a larger part of the cone or larger cones, which is similar to what is shown for SWS2B. The consequences this may have for vision is currently not known. In similar to RH2A-1, we could not identify any topographic variation in SWS2 expressing cones in adult cod. However in contrast to larvae, both of the subtypes are expressed at regular intervals, with RH2A-1 expressing cones in between, indicating an organization of cone opsins into a regular mosaic pattern. The organization of a regular mosaic pattern together with the appearance of rod at the time of metamorphosis, has been described in a number of species, and most likely improves resolution and sensitivity in the juvenile fish [66].

The opsin repertoire of a species is a result of retention and loss of ancestral forms which have undergone a number of duplications where the favourable forms have been retained in the genome. In similar to other teleosts, tandem duplications seem to have produced multiple paralogs within each subfamily of cod SWS2 and RH2A opsins. Duplicated cone opsins sensitive to wavelengths outside the species photic environment are more likely to be lost while others may increase the fidelity in the photic environment it resides and will be retained. Cod has favoured expression of blue and green sensitive cones, while opsin subfamilies sensitive towards the most extreme parts of the light field has been lost in the cod lineage. The genomic organization of opsins in tandem repeats pose question as to how the array of opsins are differentially regulated in response to changes in ecology, and also in terms of ontogenesis. Our knowledge is still limited what concerns the ecological effect on ontogenetic and spatial organization of visual pigments in teleosts, and even less is known on how this affects fish behaviour. The future use of comparative studies of species adapted to different light conditions and with different developmental strategies, together with the molecular information on visual opsins, will aid in our understanding of the role of multiple visual opsins.

## Supporting Information

S1 Fig **Alignment of RH2A deduced amino acid sequences.**
(TIF)

S2 Fig **Alignment of SWS2 deduced amino acid sequences.**
(TIF)

S3 Fig **Reverse transcription-polymerase chain reaction (RT-PCR) of visual opsins from three developmental stages of cod.**
(TIF)

S4 Fig **Nucleotide alignment of cod RH2A opsins and area used for** ***in situ*** **probe synthesis.**
(TIFF)

S1 Table **Primers used in PCR for cloning and RNA probe design for** ***in situ*** **hybridization.**
(DOCX)
