# Supplementary figures and images for: Molecular Evidence that Only Two Opsin Subfamilies, the Blue Light- (SWS2) and Green Light-Sensitive (RH2), Drive Color Vision in Atlantic Cod (Gadus morhua)
Source: PLoS One. 2014 Dec 31;9(12):e115436. doi: 10.1371/journal.pone.0115436 (PMC4281148; doi:10.1371/journal.pone.0115436)

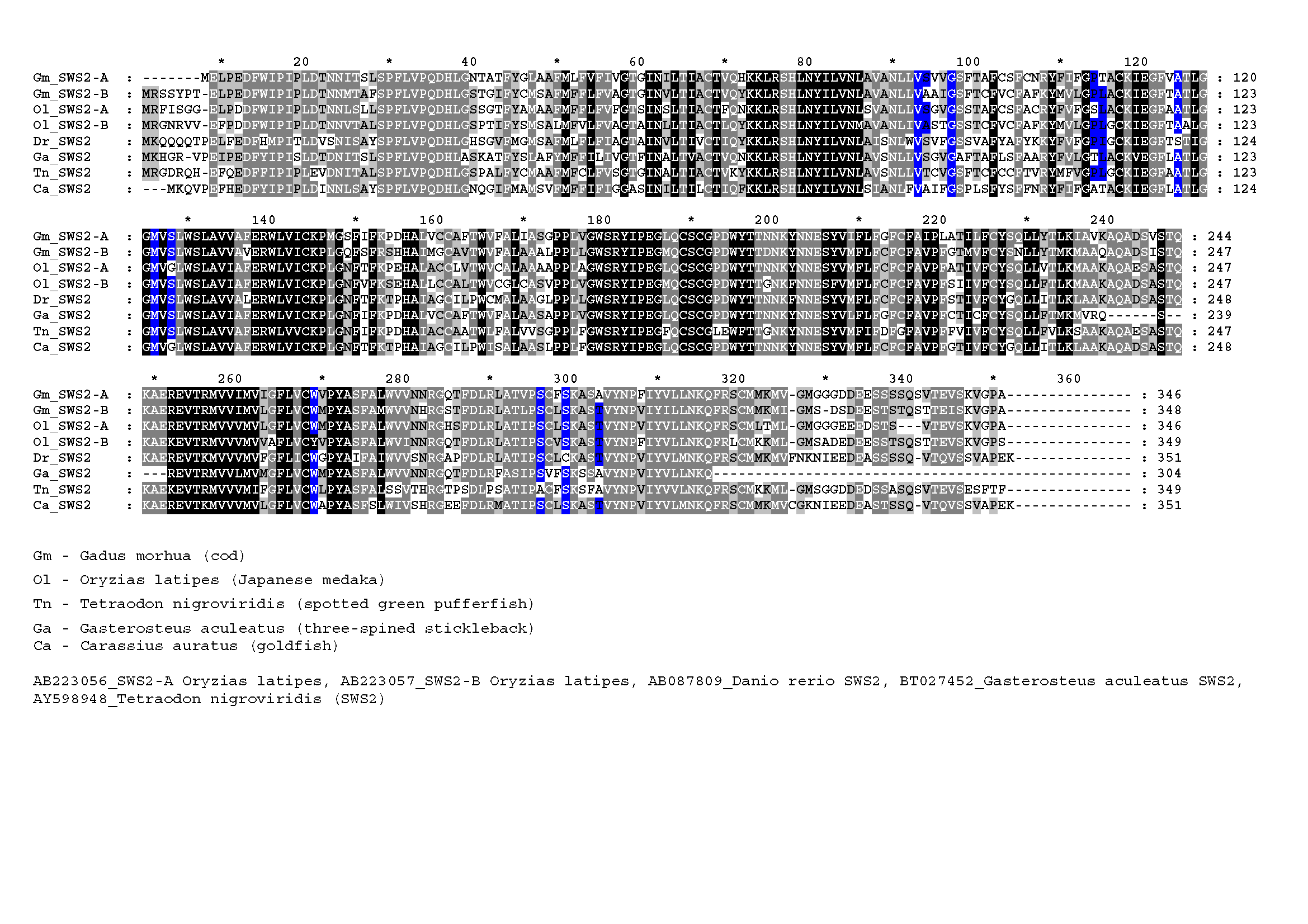

Supplement: S1 Fig — Alignment of RH2A deduced amino acid sequences. (TIF) [file pone.0115436.s001.tif]

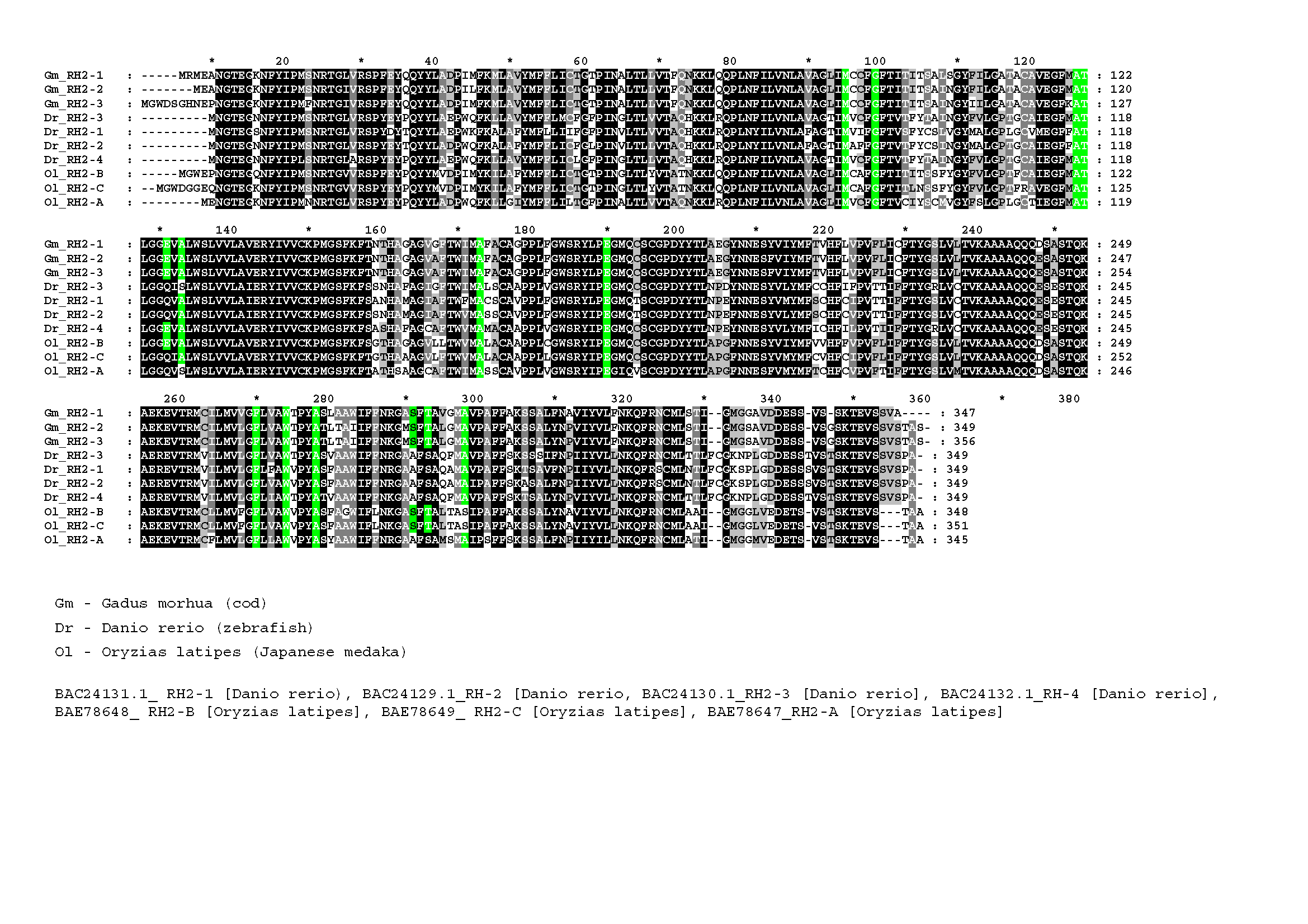

Supplement: S2 Fig — Alignment of SWS2 deduced amino acid sequences. (TIF) [file pone.0115436.s002.tif]

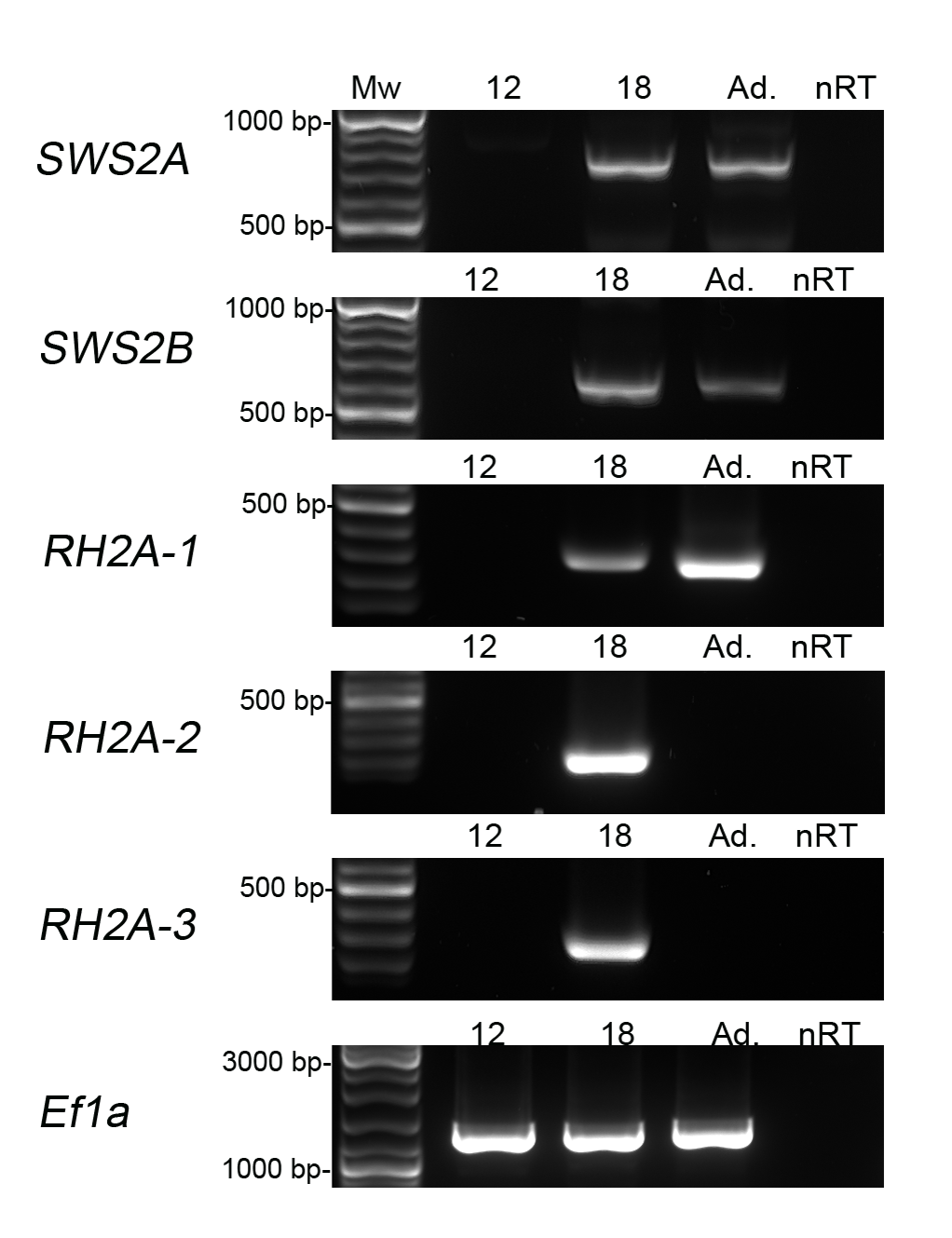

Supplement: S3 Fig — Reverse transcription-polymerase chain reaction (RT-PCR) of visual opsins from three developmental stages of cod. (TIF) [file pone.0115436.s003.tif]

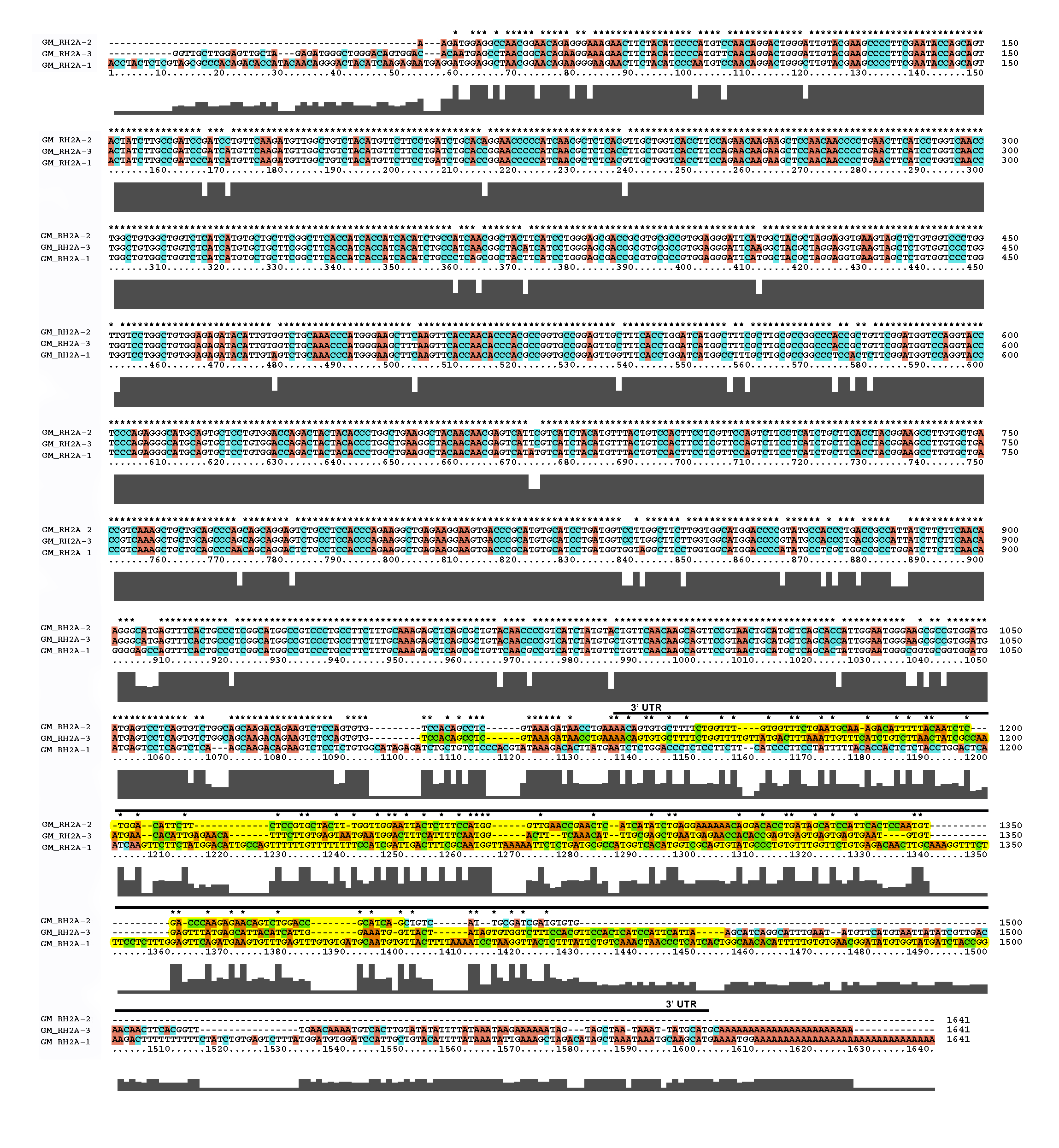

Supplement: S4 Fig — Nucleotide alignment of cod RH2A opsins and area used for in situ probe synthesis. (TIFF) [file pone.0115436.s004.tiff]
