# Supplementary material for: Molecular Evidence that Only Two Opsin Subfamilies, the Blue Light- (SWS2) and Green Light-Sensitive (RH2), Drive Color Vision in Atlantic Cod (Gadus morhua)
Source: PLoS One. 2014 Dec 31;9(12):e115436. doi: 10.1371/journal.pone.0115436 (PMC4281148; doi:10.1371/journal.pone.0115436)
Supplement: S1 Table — Primers used in PCR for cloning and RNA probe design for in situ hybridization. (DOCX) [file pone.0115436.s005.docx]

| Gene: | GenBank accession number: | Primers for PCR and *in situ* RNA probe: | Sequence (5’-3’): | Product size,  base pairs: |
| --- | --- | --- | --- | --- |
| RH2A-1 | AF385824 | *GM1_RH2-1_Fwd1*  *GM_RH2-1_Rev1* | *GTTCTTCTATGGACATTGCCAG*  *CATACCACATATCCGTTCACAC* | *286 bp* |
| RH2A-2 | KJ572530 | *GM_RH2-2_Fwd2*  *GM_RH2-2_Rev1*  *GM_RH2-2_Fwd2*  *GM_RH2-2_Rev1* | *CAACAGGACTGGGATTGTACGAAG*  *GAACAGTCTGGACCGCATCAG*  *CTGGTTTGTGGTTTCTGAATGC*  *CTGATGCGGTCCAGACTGTTC* | 1216 bp  *187 bp* |
| RH2A-3 | KJ572531 | *GM_RH2-3_Fwd2*  *GM_RH2-3_Rev1*  *GM_RH2-3_Fwd1*  *GM_RH2-3_Rev1* | *GCTGGGACAGTGGACACAATG*  *CCACGTTCCACTCATCCATTCATT*  *CCACAGCCTCGTAAAGATAACC*  *AATGAATGGATGAGTGGAACGTGG* | 1330 bp  *281 bp* |
| SWS2a | AF385822 | *GM_B2a__Fwd1*  *GM_B2a_Rev2* | *AGGAAGTTCCGTCCACCTCTC*  *ATGATAGGGTTATAGACTGCGGAGG* | 719 bp |
| SWS2b | KJ572532 | *GM_B2b_Fwd2*  *GM_B2b_Rev2* | *CGCTCGCACCTCAACTACATCCT*  *ACCACCATCCTGGTAACCTCCCT* | 560 bp |

**Table S1.** **Primers used in PCR for cloning and RNA probe design for *in situ* hybridization.** Primers written in italics were used for in situ RNA probe synthesis. For SWS2a and –b the same primers were used for PCR and cloning as for RNA probe synthesis. For all RNA probe primers, the T3 and T7 promoter sequence is not shown, see *2*. *Material and methods* part for further details.
